# Anxiety, depression, and lower quality of life among Danish women with polycystic ovarian syndrome

**DOI:** 10.1093/eurpub/ckac131.500

**Published:** 2022-10-25

**Authors:** V Abdelkarim, S Thapa

**Affiliations:** 1 Public Health, University of Southern Denmark, Odense, Denmark; 2 Research Unit of General Practice, University of Southern Denmark, Odense, Denmark

## Abstract

**Background:**

Polycystic Ovarian Syndrome (PCOS) is the most frequent hormonal/endocrine disorder in women of reproductive age and is one of the most common causes of infertility. Approximately 20% of Danish women of reproductive age are diagnosed with PCOS, and yet only a little attention has been paid to the psychological symptoms appearing in women with PCOS. The present study investigated anxiety, depression, and lower quality of life among Danish women of reproductive age who are diagnosed with PCOS.

**Methods:**

This was a cross-sectional survey study among 326 Danish women who self-reported as being diagnosed with PCOS. The Hospital Anxiety and Depression Scale (HADS) was used to measure anxiety and depression, and the PCOS-QOL scale was used to measure the quality of life. Chi-square tests and logistic regression analyses were used for analyzing data.

**Results:**

Sixty-six percent of women were found to have anxiety (n = 216), 41% of women had depression and 70% of women had low quality of life The most frequently reported symptoms were irregular menstrual cycle (86%), overweight (73%), and mood swings (65%), and irregular menstrual cycle, overweight and infertility were the were perceived of having higher severity. After adjusting for age, ethnicity, education, and income, the severity of symptoms, namely irregular menstrual cycle, infertility, overweight, and hirsutism, were independently and positively associated with depression, and severity of symptoms, namely irregular menstrual cycle, mood swings, and hirsutism, was independently and positively associated with anxiety. Lower quality of life was independently associated with both anxiety and depression.

**Conclusions:**

Women with PCOS are at higher risk of anxiety, depression, and consequently, lower quality of life. Clinicians and other healthcare professionals should pay attention to the importance of mental health support in women with PCOS and work towards promoting it.

**Key messages:**

• Women with PCOS are at higher risk of anxiety and depression, and lower quality of life.

• Health care professionals working with women with PCOS should be aware of this and be prepared to offer necessary support.

